# Special Issue “Disaster Risk Reduction and Climate Change Adaptation: An Interdisciplinary Approach”

**DOI:** 10.3390/ijerph20032641

**Published:** 2023-02-01

**Authors:** Mikio Ishiwatari, Daisuke Sasaki

**Affiliations:** 1 Graduate School of Frontier Sciences, The University of Tokyo, Kashiwa 277-8563, Japan; 2 International Research Institute of Disaster Science, Tohoku University, Sendai 980-8577, Japan

The UN member states adopted three international agreements for the post-2015 agenda: the Sendai Framework for Disaster Risk Reduction 2015–2030, the Paris Agreement of the United Nations Framework Convention on Climate Change, and the 2030 Agenda for Sustainable Development. Climate change is exacerbating disaster risks worldwide, forcing countries to enhance disaster reduction measures. Approaches geared toward adapting to climate change involve a wide range of measures that reduce disaster risks [1]. Interdisciplinary approaches to climate change adaptation (CCA) and disaster risk reduction (DRR) could help make society more resilient to various shocks and multi-hazards and help achieve the three global agendas mentioned above. Developing interdisciplinary approaches involves integrating multiple disciplines and concepts. This is because disaster risks vary by risk factors, people’s perceptions, spatial scales, development stages, and region [2]. Integrating the DRR and CCA approaches is challenging because experts and researchers have engaged with them separately [3]. Informed policymaking requires climate and socio-economic data as well as evidence of approaches’ effectiveness, something of which developing countries do not have enough [4].

This Special Issue has accepted 15 papers and the papers included cover a wide range of issues related to interdisciplinary approaches to DRR and CCA, such as methods of assessing risks and damage, people’s risk perception, financing, and policies. The findings of these studies could help promote interdisciplinary approaches at central, local, and community levels as well as internationally. We hope that this Special Issue will help accelerate research associated with the global agendas mentioned above, especially the SFDRR, which is due to undergo a midterm review soon. 

## 1. Overview of Natural Disaster Adaptation

Jia et al. [2] reviewed recent studies on natural disaster adaptation. They found that studies primarily cover socio-economic responses for farm-scale adaptation and that studies for evaluating adaptation focus on vulnerability and not on other areas, such as resilience and countermeasures. There are research gaps in adaptive governance, lifestyle and behavior changes, and innovative financing mechanisms. Moreover, some papers in this Special Issue cover people’s perceptions leading to behavior change and financing of DRR, but not governance. Future studies should cover risk governance issues. 

## 2. Risk and Damage Assessment to Mitigate Damage

Formulating evidence-based policies requires data-based analysis. The risks of extreme events provide fundamental information for formulating DRR and CCA policies. Two papers within this Special Issue assessed the risks caused by extreme temperatures in China. Shi and Ye [5] analyzed the temporal and spatial variation of extreme temperatures from 1970 to 2014 in the Yangtze River Basin, China. Indices show a decreasing trend for extreme cold temperatures and an increasing trend for extreme warm temperatures. In addition to climate change, rapid development and urbanization from the 1980s may contribute to abrupt changes in extreme temperature indices starting in the same decade. Ma et al. [6] assessed the risks of high-temperature disasters affecting kiwifruit in Shaanxi Province, China. They developed models that can identify suitable areas for producing kiwifruit and areas at risk of high-temperature disasters.

Guo et al. [7] analyzed drought vulnerability in China and revealed that the vulnerability of agriculture to drought has decreased since the 1970s. The northwest and southwest regions’ vulnerability is more severe than that of other regions. 

Improving the damage assessment process following disasters could strengthen recovery efforts. Providing accurate damage information could assist decision-makers to undertake scientifically based response and rehabilitation. Two papers in this Special Issue proposed methods of estimating damage following disasters. Li et al. [8] proposed a rapid estimation method of earthquake fatality by combining physical simulations and empirical statistics in China. Zheng et al. [9] studied excess mortality of indirect deaths caused by the Great East Japan Earthquake and Tsunami in 2011 and found that the government underreported indirect deaths. Indirect deaths are caused by factors indirectly related to disasters, such as illness deterioration due to difficult conditions while evacuating, increased stress due to drastic changes in living conditions, and suicides among evacuees. They estimated that the government had underreported 873 deaths.

## 3. People’s Risk Perception for Changing Behavior

As risk perception determines people’s protective behavior, understanding how people’s risk perception affects their behavior is useful for formulating policies to encourage people to change their behavior to reduce risks. Regarding this, two studies included in this Special Issue reached different conclusions. Wu et al. [10] analyzed people’s risk perception in at-risk areas in Sichuan Province, China. They found a positive correlation between people’s risk perception and willingness to evacuate and a negative correlation with the population at risk. Lestari et al. [11] asserted the opposite conclusion. They examined the relationships between people’s initial protection behavior, evacuation behavior, concern over the possibility of a tsunami, and natural-hazard-triggered technological (Natech) situations in an earthquake in Indonesia. The results of their study did not support the hypothesis that higher risk perception is associated with evacuation behavior or that immediate evacuation is related to foreseeing cascading sequential consequences, contrary to the existing literature. 

Two studies examined people’s perceptions related to the COVID-19 pandemic and they propose policies for managing the pandemic. These studies quantitatively clarified key factors toward realizing evidence-based policymaking for managing pandemics. Sasaki et al. [12] investigated people’s perception of well-being during the COVID-19 pandemic in Japan and advocated that the government should pay more attention to single-person households affected by the COVID-19 pandemic to improve their well-being. Pelupessy et al. [13] analyzed people’s perceptions of COVID-19 risk in Greater Jakarta, Indonesia. Individual-level perceptions affect protection behavior at a family level against COVID-19. Thus, the results suggested that improving individual-level perceptions could strengthen family-level responses to the pandemic.

## 4. Financing Investment and Policy Formulation Based on Evidence

Several studies within this Special Issue analyzed the DRR and CCA approaches, investigated capacities, and recommended policies based on evidence. Shimada [14] analyzed the impact of climate–natural disasters on economic and social variables and the impact of international aid in Africa. The study revealed that natural disasters affect economic growth, agriculture, poverty, and cause armed conflicts. In particular, droughts were the main cause of negative impacts. Although international aid had positive effects, these effects were insignificant compared to the negative impacts of natural disasters. Moreover, cereal food aid had a negative crowding-out effect on cereal production.

Ishiwatari and Sasaki [15] examined the factors affecting investments in flood protection by analyzing investment trends over a 150-year period in Japan and found investment cycles affected by damage. They proposed approaches to securing investments in DRR by enhancing policies, legislation, and institutions. 

Guo et al. [16] analyzed the effects of agricultural productive services on farmers’ climate-responsive behaviors in Jilin Province. It is common among maize farmers to change to appropriate varieties in accordance with the frost-free season. Agricultural productive services significantly affect climate-responsive behaviors by farmers.

Since local governments are responsible for responding to disasters and adapting to climate change on the ground, understanding their capacities and preparation is crucial for mitigating damage. Zhai and Lee [17] developed a model of evaluating disaster preparedness capability at a local level. They applied this model to a local government and for areas requiring improvement. Ramalho et al. [18] investigated adaptation processes by local governments in Portugal and found that most local governments have developed and are implementing CCA strategies. The local governments that were studied are familiar with nature-based solutions but underestimate community-based adaptation. 

## Data Availability

Not applicable.
